# METTL3 attenuates proliferative vitreoretinopathy and epithelial‐mesenchymal transition of retinal pigment epithelial cells via wnt/β‐catenin pathway

**DOI:** 10.1111/jcmm.16476

**Published:** 2021-03-23

**Authors:** Xinqi Ma, Chongde Long, Fangyu Wang, Bingsheng Lou, Miner Yuan, Fang Duan, Yao Yang, Jiaqing Li, Xiaobing Qian, Jieting Zeng, Shuibin Lin, Huangxuan Shen, Xiaofeng Lin

**Affiliations:** ^1^ State Key Laboratory of Ophthalmology Zhongshan Ophthalmic Center Sun Yat‐sen University Guangzhou China; ^2^ Center for Translational Medicine The First Affiliated Hospital Sun Yat‐sen University Guangzhou China; ^3^ Biobank of Eye State Key Laboratory of Ophthalmology Zhongshan Ophthalmic Center Sun Yat‐sen University Guangzhou China

**Keywords:** epithelial‐mesenchymal transition, m6A RNA methylation, METTL3, proliferative vitreoretinopathy, retinal pigment epithelial

## Abstract

Proliferative vitreoretinopathy (PVR) is a refractory vitreoretinal fibrosis disease, and epithelial‐mesenchymal transition (EMT) of retinal pigment epithelial (RPE) cells is the key pathological mechanism of PVR. However, few studies focused on the role of METTL3, the dominating methyltransferase for m6A RNA modification in PVR pathogenesis. Immunofluorescence staining and qRT‐PCR were used to determine the expression of METTL3 in human tissues. Lentiviral transfection was used to stably overexpress and knockdown METTL3 in ARPE‐19 cells. MTT assay was employed to study the effects of METTL3 on cell proliferation. The impact of METTL3 on the EMT of ARPE‐19 cells was assessed by migratory assay, morphological observation and expression of EMT markers. Intravitreal injection of cells overexpressing METTL3 was used to assess the impact of METTL3 on the establishment of the PVR model. We found that METTL3 expression was less in human PVR membranes than in the normal RPE layers. In ARPE‐19 cells, total m6A abundance and the METTL3 expression were down‐regulated after EMT. Additionally, METTL3 overexpression inhibited cell proliferation through inducing cell cycle arrest at G0/G1 phase. Furthermore, METTL3 overexpression weakened the capacity of TGFβ1 to trigger EMT by regulating wnt/β ‐catenin pathway. Oppositely, knockdown of METTL3 facilitated proliferation and EMT of ARPE‐19 cells. In vivo, intravitreal injection of METTL3‐overexpressing cells delayed the development of PVR compared with injection of control cells. In summary, this study suggested that METTL3 is involved in the PVR process, and METTL3 overexpression inhibits the EMT of ARPE‐19 cells in vitro and suppresses the PVR process in vivo.

## 1 INTRODUCTION

Proliferative vitreoretinopathy (PVR) is a vitreoretinal fibrosis disease, which causes repeated retinal detachment and eventually leads to blindness. PVR is characterized by the formation of proliferative membranes.^1^ Currently, surgical removal of proliferative membranes is the main treatment of PVR, which is still unable to prevent cell proliferation.^2^ The pathological response of retinal pigment epithelium (RPE) cells plays a pivotal role in the PVR process.^3^ After interacting with various cytokines, RPE cells are triggered into epithelial‐mesenchymal transition (EMT) process, and eventually convert into myofibroblasts, which become the dominant cells for contraction of proliferative membranes.^4^ Among those cytokines, transforming growth factor‐beta (TGFβ) is a pivotal growth factor known to induce EMT of RPE cells, and is present at high levels in PVR patients.^5^

The EMT of RPE cells is considered as the key pathologic mechanism of PVR.^3^ Upon the initiation of the EMT process, RPE cells lose their polarity as well as tight junctions, and acquire stronger ability of proliferation and migration, gradually evolving into mesenchymal cells in morphology and biological behaviour. During EMT process, epithelial markers including ZO‐1 are down‐regulated, while mesenchymal markers such as N‐cadherin and alpha smooth muscle actin (αSMA) are up‐regulated.^6^ As a marker of myofibroblasts, αSMA often serves as an indicator of the occurrence of EMT.^7^ Detachment of RPE cells from the basement membrane and cell migration are considered to occur in an early stage of PVR pathology, which are meditated by wnt/β‐catenin pathway. As a highly conserved pathway through evolution, wnt/β‐catenin pathway is reported to modulate tissue movement and participate in EMT process in several kinds of cells including RPE cells.^1^, ^8^, ^9^, ^10^ Although the process of EMT has been investigated previously, further studies are needed to elucidate the underlying molecular regulatory mechanism.

It was reported that epigenetic modifications including DNA methylation and histone acetylation, could regulate the EMT of RPE cells.^11^, ^12^ However, recent studies showed that epigenetic modifications of mRNA also play a key role in modulating several biological processes, and whether it also takes a part in EMT of RPE cells remained unknown. N6‐methyladenosine (m6A) is the most common epigenetic modification of mRNA, which mediates more than 80% of RNA methylation.^13^, ^14^ The m6A modification has reversible and dynamic properties, whose balance is orchestrated by three different types of protein complex including ‘writer’ methyltransferases, ‘eraser’ demethylases, and ‘reader’ proteins that recognize m6A‐modified mRNA sites.^15^ However, as the critical catalytic subunit in m6A ‘writer’ methyltransferases, METTL3 plays a key role in generating m6A.^16^, ^17^ In recent years, scientists have found that METTL3 plays different roles in suppressing or promoting EMT in different cancer cells,^18^, ^19^, ^20^ which indicates that the regulatory effect of METTL3 is cell‐type specific.^21^, ^22^ However, to further understand the regulatory mechanism of PVR, it is essential to clarify the role of METTL3 in RPE cells. The crosstalk between METTL3 and the TGFβ signalling pathway in cancer cells also implies a correlation between METTL3 and EMT.^23^, ^24^, ^25^ And it is a remarkable fact that in addition to tumour metastasis, EMT also occurs during organ fibrosis, wound healing and embryonic development.^26^, ^27^ However, little is known about the role of METTL3 in these processes, and even less is known about the relationship between METTL3 and proliferative vitreoretinopathy (PVR).

In this study, we found differences in METTL3 expression between human PVR membranes and normal RPE layers, and then we investigated the effect of METTL3 overexpression on EMT of ARPE‐19 cells and wnt/β‐catenin pathway. Additionally, to determine the role of METTL3 in the EMT of ARPE‐19 cells in vivo, we injected cells overexpressing METTL3 into rat vitreous cavities and assessed their capabilities to form PVR membranes. Our data uncovered the critical function of METTL3 in regulation of proliferative vitreoretinopathy, and the elucidation of this regulatory mechanism will provide new ideas for the treatment of PVR.

## 2 MATERIALS AND METHODS

### 2.1 Human tissue collection and immunofluorescence analysis

The study protocol involving human patients was approved by the Ethics Committee of Zhongshan Ophthalmic Center (Guangzhou, China), in agreement with the Declaration of Helsinki, and written informed consent was obtained from each subject. ERMs and SRMs were obtained from PVR patients who underwent vitreoretinal surgery. Surgery for all patients strictly followed the indications of operation. RPE layers were obtained from normal donor eyes from Guangdong Eye Bank (Guangzhou, China). Characteristics of the patients/subjects were listed in Table 1.

**TABLE 1:** Characteristics of the patients/subjects for immunofluorescence and qRT‐PCR

Figures	Age(y)	Sex	Diseases	Stages of PVR	Tissues
1A	19	Male	RRD	D2	ERM
	28	Female	RRD	D1	SRM
1B	62	Male	RRD	D1	ERM
	40	Female	RRD	D1	SRM
1C、1D、1E	47	Male	Normal control		RPE layer^a^
	27	Male	Normal control		RPE layer^a^
	26	Male	Normal control		RPE layer^a^
	46	Male	Normal control		RPE layer^a^
	28	Female	Normal control		RPE layer^a^
	41	Female	Normal control		RPE layer^a^
	44	Female	Normal control		RPE layer^a^
	71	Male	RRD	C1	ERM
	52	Male	RRD	D1	ERM
	18	Male	RRD	D2	ERM
	33	Male	RRD	D2	ERM
	53	Male	RRD	C1	ERM
	70	Male	RRD	D2	ERM
	68	Male	RRD	C1	ERM
	60	Male	RRD	C1	ERM
	48	Male	RRD	C2	ERM

Abbreviations: ERM, epiretinal membrane; RRD, rhegmatogenous retinal detachment; SRM, subretinal membrane.

^a^ Donor eyes from Guangdong Eye Bank (Guangzhou, China).

Upon dissection, the specimen was fixed by immediately immersing in 4% paraformaldehyde (PFA; pH7.4) for 24 h. The fixed specimen was embedded in optimum cutting temperature compound (OCT), frozen and sliced into 8‐ μm thick sections, and those sections were stored at −80°C. To perform immunofluorescence staining, the sections were thawed at room temperature for 2 h, washed with phosphate‐buffered saline (PBS; pH7.4), then punched with 0.3% Triton (Thermo Fisher Scientific, Rockford, US) for 10 min. Then, the sections were blocked with 10% normal goat serum at room temperature for 90 min and incubated with a mixture of primary antibodies, including pan‐cytokeratin antibody (1:200; Thermo Fisher Scientific, Rockford, US), αSMA antibody (1:200; Sigma‐Aldrich Corp.), and METTL3 antibody (1:500; Abcam, Cambridge, UK) at 4°C overnight. Subsequently, the sections were incubated with a mixture of secondary antibodies, including goat anti‐mouse Alexa Fluor^®^ Plus 555 and donkey anti‐rabbit Alexa Fluor^®^ Plus 488 (1:500; Thermo Fisher Scientific, Rockford, US), at 37°C for 90 min. The nuclei were labelled with DAPI (Thermo Fisher Scientific, Rockford, US), and images were taken under a confocal laser scanning microscope (Carl Zeiss, Oberkochen, Germany). Sections incubated in the buffer containing no primary antibodies were used as a negative control to examine the specific staining of target proteins.

### 2.2 Cell culture and transfection

A human retinal pigment epithelial cell line (ARPE‐19) was purchased from American Type Culture Collection (ATCC, Manassas, VA, US) and cultured in Dulbecco's Modified Eagle's Medium/Nutrient Mixture F‐12 (DMEM/F‐12; Gibco, Grand Island, NY, US) supplemented with 10% fetal bovine serum (FBS; Gibco, Grand Island, NY, US) at 37°C in a humid atmosphere containing 5% CO2. Recombinant human TGFβ1 was purchased from Novoprotein. SKL2001, the agonist of wnt/β‐catenin pathway, was purchased from MedChemExpress.

The pCDH‐Vec control vectors and pCDH‐METTL3 overexpression vectors as well as pLKO.1‐TRC‐Vec control vectors and pLKO.1‐TRC‐shMETTL3 knockdown vectors used for stable transfection were kindly provided by Prof. Shuibin Lin.^18^ Plasmids were transfected into ARPE‐19 cells using lentivirus, as described previously. Stably transformed ARPE‐19 cells were selected by selection on media containing 2  μg/ml puromycin (Solarbio Life Science, CN) for 48 h.^28^

### 2.3 Measurement of m6A abundance

The level of m6A in ARPE‐19 cells was detected using the EpiQuik m6A RNA Methylation Quantification Kit (Fluorometric) (Epigentek, Farmingdale, US) according to the manufacturer's instructions. Briefly, total RNA was bound to the bottom of the 96‐well plate with the Binding Solution (BS) and incubated at 37°C for 90 min. Then, samples were captured with the Capture Antibody (CA; 1:1000), by incubation at room temperature for 60 min. Subsequently, the Detection Antibody (DA; 1:2000) was added to the plate. After incubation at room temperature for 30 min, the Enhancer Solution (ES) was added to the plate, and the samples were incubated at room temperature for 30 min, Relative Fluorescence Units (RFU) in Fluoro‐Development Solution were measured using the SpectraMax® fluorescence microplate reader at 530 and 590 nm excitation and emission wavelengths, respectively. The total m6A level was calculated based on the standard curve using the following equation:

$$\text{m6A(ng)}=\frac{\text{Sample RFU}‐\text{NC RFU}}{\text{Slope}}$$

$$\text{m6A\%}\;=\frac{\text{m6A Amount}\;(\text{ng})}{S}\times 100\%$$

where *S* is the amount of input sample RNA in ng.

### 2.4 Immunofluorescence staining for cells

Immunofluorescence staining for cells was performed as described previously.^29^ The following was used as the primary antibody: METTL3 (1:500; Abcam, Cambridge, UK). The following was used as the secondary antibody: anti‐rabbit Alexa Fluor^®^ Plus 488 (1:500; Thermo Fisher Scientific, Rockford, US).

### 2.5 Apoptosis, cell cycle detection and MTT assay

Apoptosis, cell cycle detection and MTT assay were performed as described previously ^29^, ^30^ using the Multicaspase Kit (Millipore Corporation, Hayward, US), Cell Cycle Detection Kit (BD Pharmingen, San Diego, US) and Thiazolyl Blue Tetrazolium Bromide (MTT, Sigma‐Aldrich Corp.).

### 2.6 Transwell assay

The transwell assay was conducted using a 24‐well transwell plate with a polycarbonate membrane (pore size = 8 μm; Corning Incorporated, NY, US). ARPE‐19 cells were digested into a cell suspension of 200 μl without FBS and seeded in the upper chambers at a density of 8 × 10^4^ cells. The lower chambers were filled with 500μl of ARPE‐19 culture medium containing 20% FBS. After incubation for 12 h and 24 h, cells that passed through the membrane were fixed to the membrane with methanol (Boster Biological Technology, CN) and then stained with crystal violet (Solarbio Life Science, CN) at room temperature for 15 min. Images were captured using a light microscope (Carl Zeiss, Oberkochen, Germany). Cell migration ability was assessed based on the cell numbers that passed through the polycarbonate membrane, which were analysed and calculated using the Image J software.

### 2.7 Wound healing assay

ARPE‐19 cells were seeded in 6‐well plates. When the cell density reached 80%, scratches were made by a p200 pipette tip. Cells were observed and photographed under an optical microscope at 0, 24 and 48 h. The cell migration ability was reflected by the wound healing area, which was analysed using the Image J software and calculated as follows:

$$\text{Wound healing area}\;\left(\%\right)=\;\frac{A_{0}‐A_{24/48}}{A_{0}}\times 100\%$$

**A_0_** is the area of the wound at 0 h, and **A_24/48_** is the area of the wound at 24/48 h.

### 2.8 Western blot analysis

Western blot analysis was performed as described previously,^31^ The following are used as the primary antibodies: METTL3 (1:1000; Abcam, Cambridge, UK), N‐cadherin (1:10 000; Abcam, Cambridge, UK), αSMA (1:500; Sigma‐Aldrich Corp), ZO‐1 (1:500; Invitrogen, Carlsbad, CA, US), MMP9 (1:500; Abcam, Cambridge, UK), pGSK3β (phospho Ser9) (1:1000, Immunoway Biotechnology, Plano, US), GSK3β (1:1000, Immunoway Biotechnology, Plano, US), cyclinD1 (1:1000, Immunoway Biotechnology, Plano, US), β‐catenin (1:1000, Cell Signaling Technology), β‐actin (1:1000, Cell Signaling Technology) ，β‐tubulin (1:1000, Cell Signaling Technology) and GAPDH (1:1000; Cell Signaling Technology). The following were used as secondary antibodies: goat anti‐rabbit IgG H&L (HRP) pre‐adsorbed antibody (1:5000; Abcam, Cambridge, UK) and goat anti‐mouse IgG H&L (HRP) pre‐adsorbed antibody (1:5000; Abcam, Cambridge, UK).

### 2.9 qRT‐PCR

Quantitative real‐time PCR was performed as described previously ^31^ using the follow primers: METTL3 (forward: 5’‐CAAGCTGCACTTCAGACGAA‐3’and reverse: 5’‐GCTTGGCGTGTGGTCTTT‐3’), αSMA (forward: 5’‐GGCTGTTTTCCCATCCATTGT‐3’ and reverse: 5’‐TCTTTTGCTCTGTGCTTCGT‐3’). Expression data were normalized to the geometric mean of housekeeping gene GAPDH (forward: 5’‐GAGTCCACTGGCGTCTTCAC‐3’ and reverse: 5’‐GTTCACACCCATGACGAACA‐3’).

### 2.10 PVR induction and identification in rat eyes

All animal experiments in this study have been approved by the Animal Care and Use Committee of Sun Yat‐sen University and strictly adhered to the National Institutes of Health (NIH) guide for the care and use of laboratory animals. The study was carried out as described previously,^32^, ^33^ with slight modifications. Briefly, 46 Brown Norway (BN) 6‐8‐week‐old male rats (weight = 200 ± 10 g) of SPF grade were randomly divided into three groups, 20 rats in group 1, 20 rats in group 2 and 6 rats in group 3. The vitreous cavity of the right eye of each rat was injected either with ARPE‐19 cells (1 × 10^5^ per μl in PBS [pH 7.4]) overexpressing METTL3 or control vector cells or with an equal volume of PBS (pH 7.4). The experimental animals were anaesthetized with chloral hydrate, then an antibiotic and a topical anaesthetic were applied to the right eye after pupillary dilation. Subsequently, a 33‐gauge insulin needle was used to pierce through the sclera, and 3 μl of cell suspension or PBS was injected into the vitreous cavity with a 33G Hamilton syringe fitted with a 33‐gauge microneedle. After operation, the right eye was coated with an antibiotic eye ointment. Vitreoretinal conditions of all injected eyes were evaluated and graded by two masked observers at 1, 3, 7, 10 and 14 days after the operation, and fundus photographs were acquired using the retinal imaging system (Phoenix Micron IV). Because one rat injected with the control vector cells developed cataract after the injection, this rat was not involved in fundus observation. The identification of rat PVR stages (0‐3) follows the rat PVR grading scale (Behar‐Cohen et al, 2000): 0, no proliferative response; 1, intravitreal proliferation; 2, epiretinal membrane formation with retinal folds; 3, white dense membrane covering the retina, with retinal folds and localized retinal detachments with or without a localized posterior capsular cataract.^34^

### 2.11 Histological detection and immunohistochemistry of rat retina

All rats were sacrificed on day 14 by cervical dislocation. Eyeballs of the dead rats were removed, fixed in 10% neutral buffered formalin (NBF; Thermo Fisher Scientific, Rockford, US) for 24 h and dehydrated using reagent alcohol by gradient. The dehydrated samples were embedded in paraffin and sliced to a thickness of 5 μm. Haemotoxylin and eosin (H&E) staining was performed for the histological analysis of the retina and proliferative membranes, and immunofluorescence of αSMA was carried out to further identify proliferative membranes at the molecular level.

### 2.12 Statistical analysis

Statistical analysis was carried out using IBM SPSS 21.0. All data are presented as mean ± standard deviation (SD) of at least three independent experiments unless otherwise specified. Unpaired two‐tailed Student's t*‐*test was used for comparison between two groups, and one‐way ANOVA followed by Bonferroni's post hoc test was applied for multiple comparisons. Mann‐Whitney U test was used for ordinal data suitable for non‐parametric tests. *P <.05, **P <.01, ***P <.001, ****P <.0001, #*P* >.05.

## 3 RESULTS

### 3.1 METTL3 showed differential expression between normal RPE layers and PVR membranes

**FIGURE 1:**
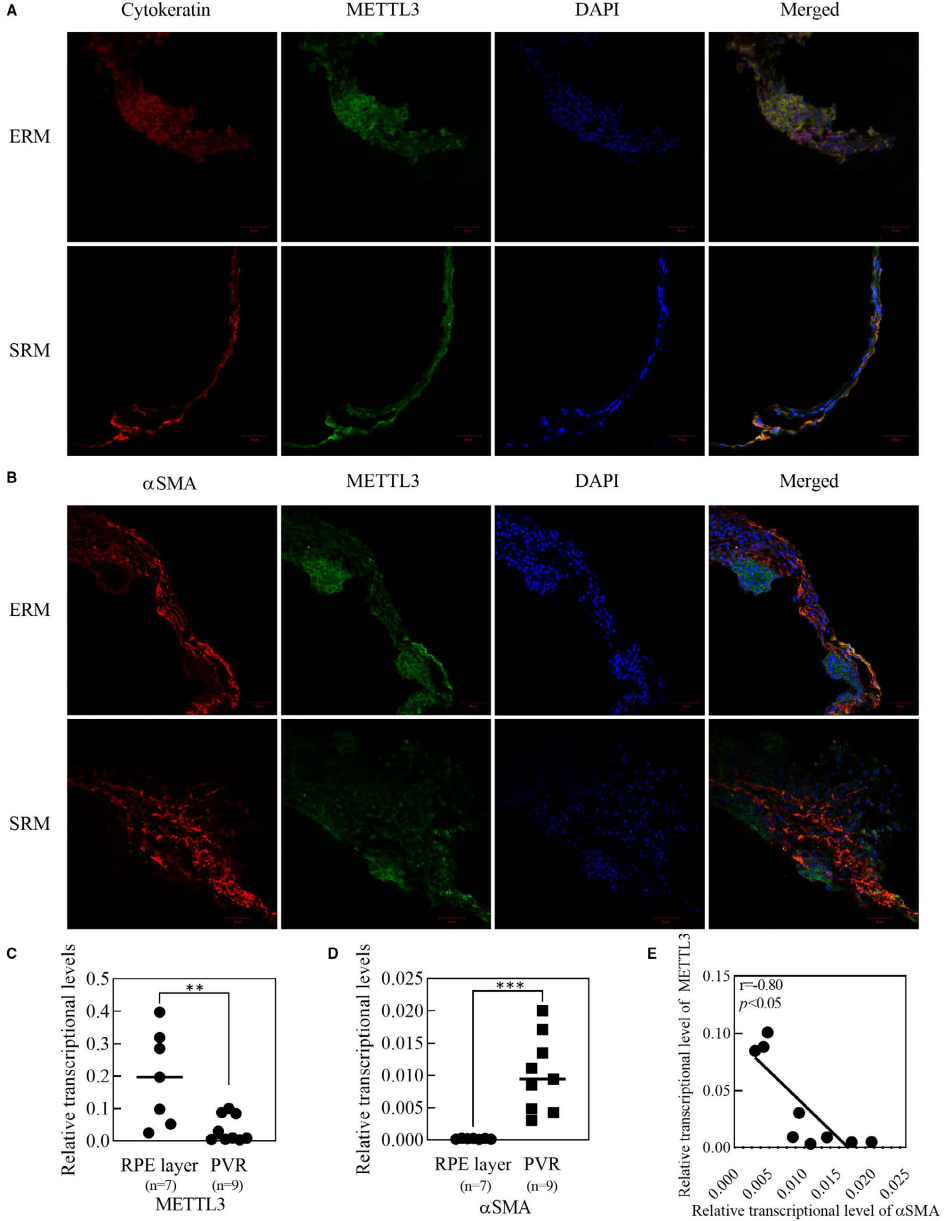
Analysis of METTL3 expression in human tissues. A, B, Immunofluorescent staining of PVR membranes with Cytokeratin‐antibody (red), αSMA‐antibody (red) and METTL3‐antibody (green). Cell nuclei were stained with DAPI (blue). Scare bar: 50 μm. C, D, Expression analysis of METTL3 and αSMA by real‐time qPCR. E, Spearman correlation analysis of METTL3 expression and αSMA expression in PVR membranes, and there was strong negative correlation between them (r = ‐0.80. *P* <.05, n = 9). Statistical significance was determined by the unpaired two‐tailed Student's t‐test (**P <.01, ***P <.001). ERM, epiretinal membrane; SRM, subretinal membrane

Immunofluorescence staining showed that METTL3 was positively expressed on PVR membranes including ERMs and SRMs, and coexisted with cytokeratin, which is a marker of RPE cells (Figure 1A). However, it seemed that the fluorescence of METTL3 was less overlapping with αSMA, which is a marker of myofibroblasts (Figure 1B). The specific staining of cytokeratin, αSMA and METTL3 was confirmed by the absence of the primary antibodies (Figure S1A). Results of qRT‐PCR suggested that in normal RPE layer, METTL3 expression was much higher compared with ERMs, while αSMA expression showed the opposite trend (Figure 1C,D). Then we analysed the relationship of expression between METTL3 and αSMA in ERMs, and it appeared that the expression of METTL3 was negatively correlated with the expression of αSMA (Figure 1E).

### 3.2 m6A abundance and METTL3 expression were down‐regulated after the EMT of ARPE‐19 cells

**FIGURE 2:**
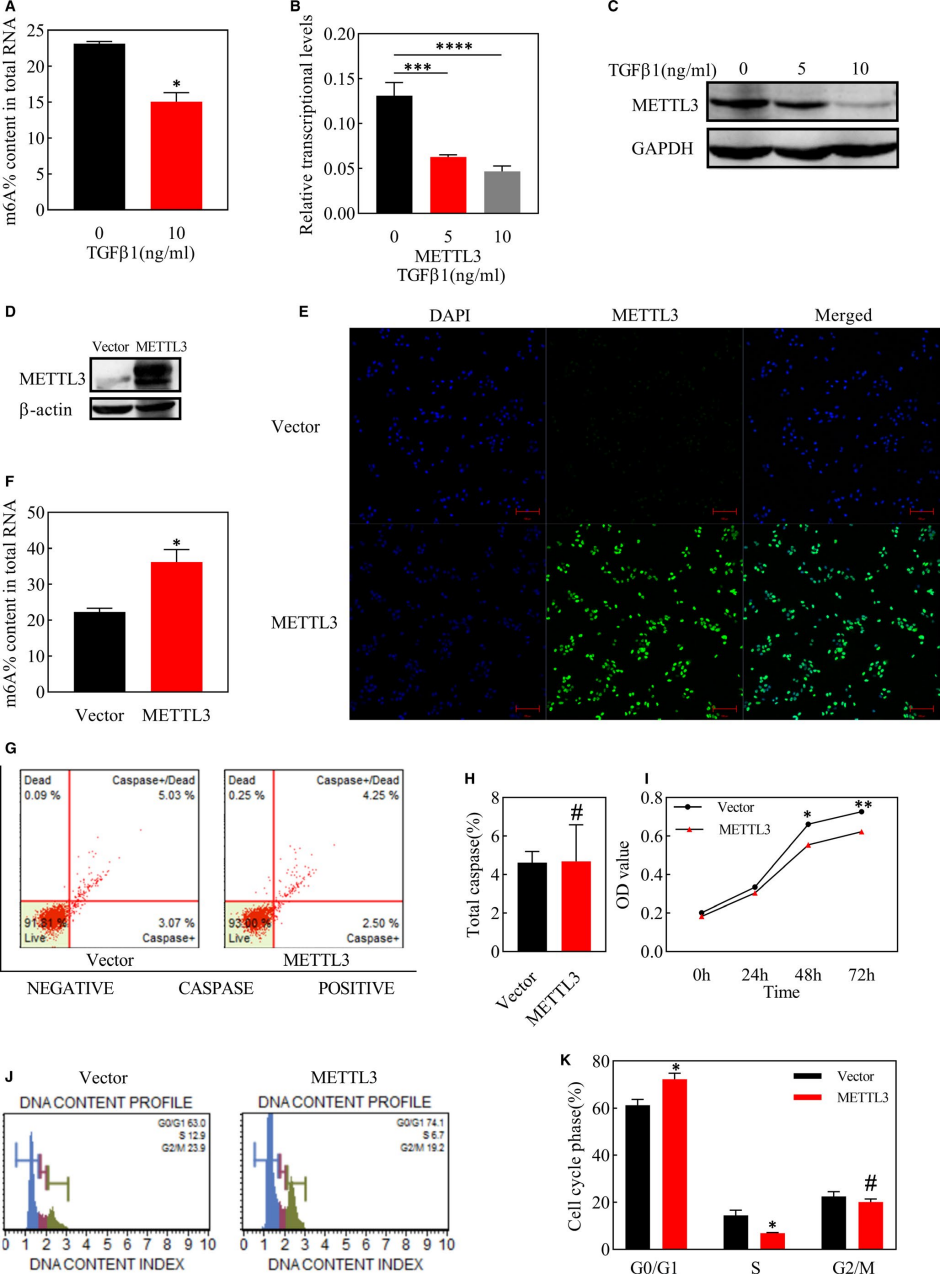
Analysis of METTL3 expression and total m6A level after EMT induction and effects of METTL3 overexpression on apoptosis, proliferation and cell cycle of ARPE‐19 cells. A, Total m6A level in ARPE‐19 cells after treated by TGFβ1 with the concentration of 10ng/ml for 48h B, C, Expression analysis of METTL3 by real‐time qPCR and western blot after treated by TGFβ1 with the concentration of 5ng/ml and 10ng/ml for 48 h. D, Western blot analysis of cells transformed with lentivirus and screened with puro for 48 h. E, Fluorescence intensity of intracellular METTL3 labelled and localized specifically by METTL3 antibody (green), as examined by confocal laser scanning microscopy. Cell nuclei were stained with DAPI (blue). Scare bar: 100 μm. F, Level of m6A in total RNA detected using the EpiQuik m6A RNA Methylation Quantification Kit (Fluorometric). G, Flow cytometry analysis of cell apoptosis at 24 h after the seeding of ARPE‐19 cells. H, Statistical analysis of the proportion of total caspase. I, Optical density (OD) values of ARPE‐19 cells in the MTT assay at 0, 24, 48 and 72 h after seeding. J, Flow cytometry analysis of cell cycle at 24 h after the seeding of ARPE‐19 cells. K, Statistical analysis of the proportion of cells in each stage of the cell cycle including G0/G1, S and G2/M. Data represent mean ± SD (n = 3). Statistical significance was determined by the unpaired two‐tailed Student's t‐test for pairwise comparisons and by one‐way ANOVA, followed by Bonferroni's post hoc test for multiple comparisons (*P <.05, **P <.01, ***P <.001, ****P <.0001, #*P* >.05)

We used TGFβ1 to induce EMT in ARPE‐19 cells and then detected total abundance of m6A, which was found to be down‐regulated after 48h of EMT induction (Figure 2A). To identify the proteins responsible for the decline in total m6A level, the expression of m6A writers (METTL3 and WTAP), eraser (ALKBH5) and reader (YTHDF2) were detected by qPCR, and the most remarkable change was observed in METTL3 (Figure 2B, Figure S1B). Meanwhile, METTL3 expression also declined in protein level after EMT, with the concentration of TGFβ1 at 10 ng/ml being more pronounced (Figure 2C).

### 3.3 METTL3 overexpression inhibited proliferation of ARPE‐19 cells through inducing G0/G1 arrest

The expression of METTL3 was significantly elevated at the protein level after lentiviral transfection with METTL3 overexpression plasmid (Figure 2D). Immunofluorescence staining showed that METTL3 was primarily expressed in the cell nucleus in both the vector group and METTL3 overexpression group (Figure 2E), and the total m6A abundance increased along with the overexpression of METTL3 (Figure 2F).

We assessed toxic effects of METTL3 overexpression on ARPE‐19 cells through apoptosis assay and observed no significant difference between the METTL3 overexpression group and the vector group (Figure 2G,H). However, MTT assay showed that METTL3 inhibited the proliferation of ARPE‐19 cells on 48h and 72h after cells inoculation (Figure 2I). Furthermore, we detected the ratio of cells in the G0/ G1 phase was higher in the METTL3 overexpression group, while the ratio of cells in the S phase showed the opposite trend, which implied that overexpressing METTL3 induced cell cycle arrest at the G0 / G1 phase, which might be partly responsible for the suppressed proliferation by METTL3 overexpression (Figure 2J,K).

### 3.4 METTL3 overexpression suppressed cell migration and TGFβ1‐induced EMT in ARPE‐19 cells

**FIGURE 3:**
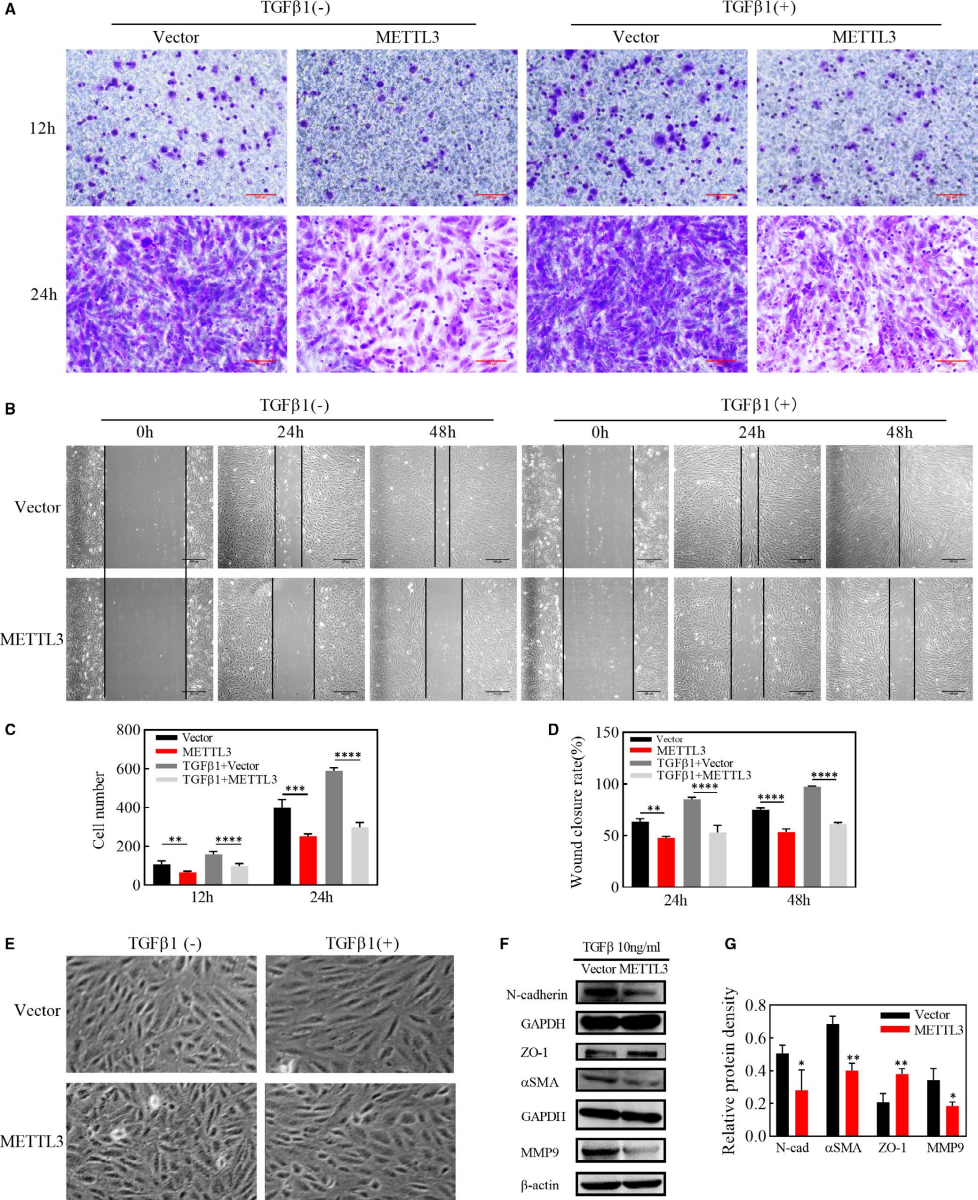
Effects of METTL3 overexpression on the EMT of ARPE‐19 cells in the presence or absence of TGFβ1. A, Migration ability of ARPE‐19 cells after seeding with or without TGFβ1 for 12 or 24 h, as measured by the transwell assay. Scale bar: 100μm. C, Statistical analysis of the number of cells that migrated through the upper chamber. B, Wound‐repair ability of ARPE‐19 cells was determined by the scratch assay with or without TGFβ1 for 0, 24 and 48 h. Scare bar: 100μm. D, Statistical analysis of the rate of wound closure. E, Cell morphology of the METTL3 overexpression and vector groups seeded with or without TGFβ1 (10 ng/ml) for 48 h. F, Western blot analysis of the epithelial marker ZO‐1 and mesenchymal markers, N‐cadherin, αSMA and MMP9, after treatment with TGFβ1 for 48 h. G, Statistical analysis of the relative protein density, which was normalized to GAPDH and β‐actin. Data represent mean ± SD (n = 3). Statistical significance was determined by the unpaired two‐tailed Student's t‐test for pairwise comparisons and by one‐way ANOVA, followed by Bonferroni's post hoc test for multiple comparisons (*P <.05, **P <.01, ***P <.001, ****P <.0001)

PVR is essentially an excessive wound‐repair response, and enhanced migration capabilities of RPE cells are vital for EMT process. We used TGFβ of 10ng/ml to induce EMT in ARPE‐19 cells and detected in transwell assay that migration speed of the METTL3 overexpression group was visibly lower than that of the vector group at 12h and 24h after cell seeding, whether in the existence of TGFβ1 or not, as shown by the number of cells migrating through the filter (Figure 3A,C). Consistent with the transwell assay, overexpressing METTL3 significantly down‐regulated the wound closure rate both before EMT and after 24h and 48h of EMT induction (Figure 3B,D), indicating that METTL3 could weaken the migration ability of ARPE‐19 cells both with or without EMT process. After EMT induction, cells were arranged more regularly in the vector group, and their morphology changed from a polygonal shape to a long spindle shape. By contrast, cells in the METTL3‐overexpression group were disorganized and maintained the primary polygonal shape (Figure 3E). However, the occurrence and progression of EMT was further verified by the down‐regulation of epithelial markers and the up‐regulation of mesenchymal markers. Western blot analysis showed that the epithelial marker ZO‐1 was up‐regulated, while the mesenchymal markers, including αSMA, N‐cadherin and MMP9 were down‐regulated in the METTL3 overexpression group (Figure 3F,G).

### 3.5 Effects of METTL3 on wnt/β‐catenin signalling pathway in EMT of ARPE‐19 cells

**FIGURE 4:**
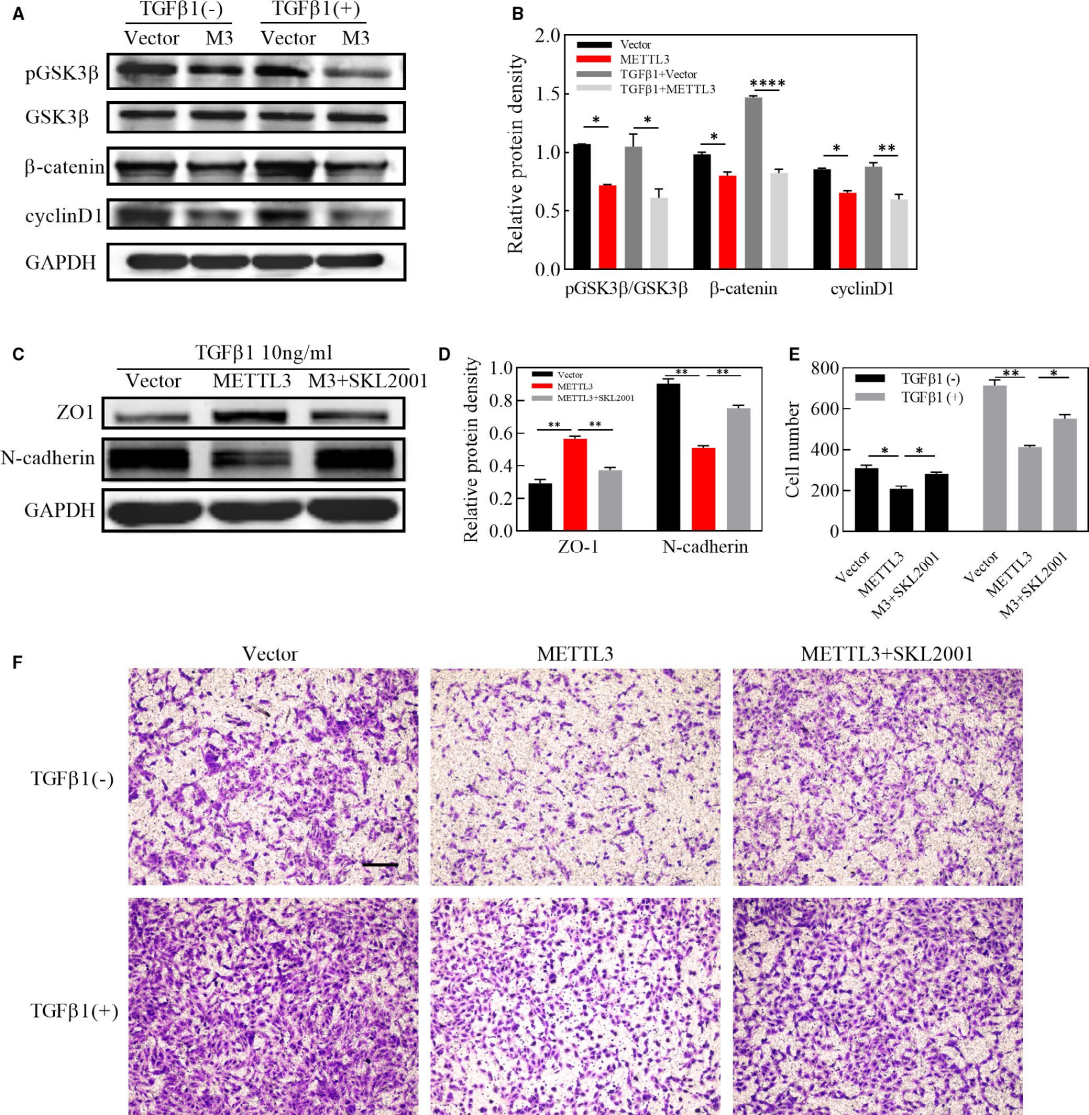
Effects of METTL3 overexpression on wnt/β‐catenin pathway and effects of activation of wnt/β‐catenin pathway on EMT process of cells overexpressing METTL3. A, Western blot analysis of the proteins of wnt/β‐catenin pathway, including pGSK3β, GSK3β, β‐catenin and cyclinD1, with or without treatment with TGFβ1 for 48 h B, Statistical analysis of the relative protein density, which was normalized to GAPDH. C, Western blot analysis of the epithelial marker ZO‐1 and mesenchymal marker N‐cadherin after treatment with TGFβ1 for 48 h. D, Statistical analysis of the relative protein density, which was normalized to GAPDH. F, Migration ability of ARPE‐19 cells after seeding with or without TGFβ1 for 24 h, as measured by the transwell assay. Scale bar: 100μm. E, Statistical analysis of the number of cells that migrated through the upper chamber. Statistical significance was determined by one‐way ANOVA, followed by Bonferroni's post hoc test for multiple comparisons (*P <.05, **P <.01, ****P <.0001)

It has been well established that wnt/β‐catenin signalling pathway is a highly conserved pathway through evolution, who plays a crucial part in cell migration. We have demonstrated that METTL3 inhibited migration of ARPE‐19 cells together with down‐regulating the expression of MMP9, the downstream gene of wnt/β‐catenin pathway. Furthermore, we explored the role of METTL3 in the wnt/β‐catenin pathway and it was indicated by western blot that the protein level of pGSK3β, β‐catenin and cyclinD1 was strikingly down‐regulated in METTL3 overexpression cells, even in the absence of TGFβ1 (Figure 4A,B). In addition, SKL2001, an agonist of wnt/β‐catenin signalling pathway, abolished the effect of METTL3 on EMT and cell migration, indicating that METTL3 exerted its regulating role through the wnt/β‐catenin pathway (Figure 4C,D,E,F).

### 3.6 Overexpression of METTL3 attenuated the development of PVR in vivo

**FIGURE 5:**
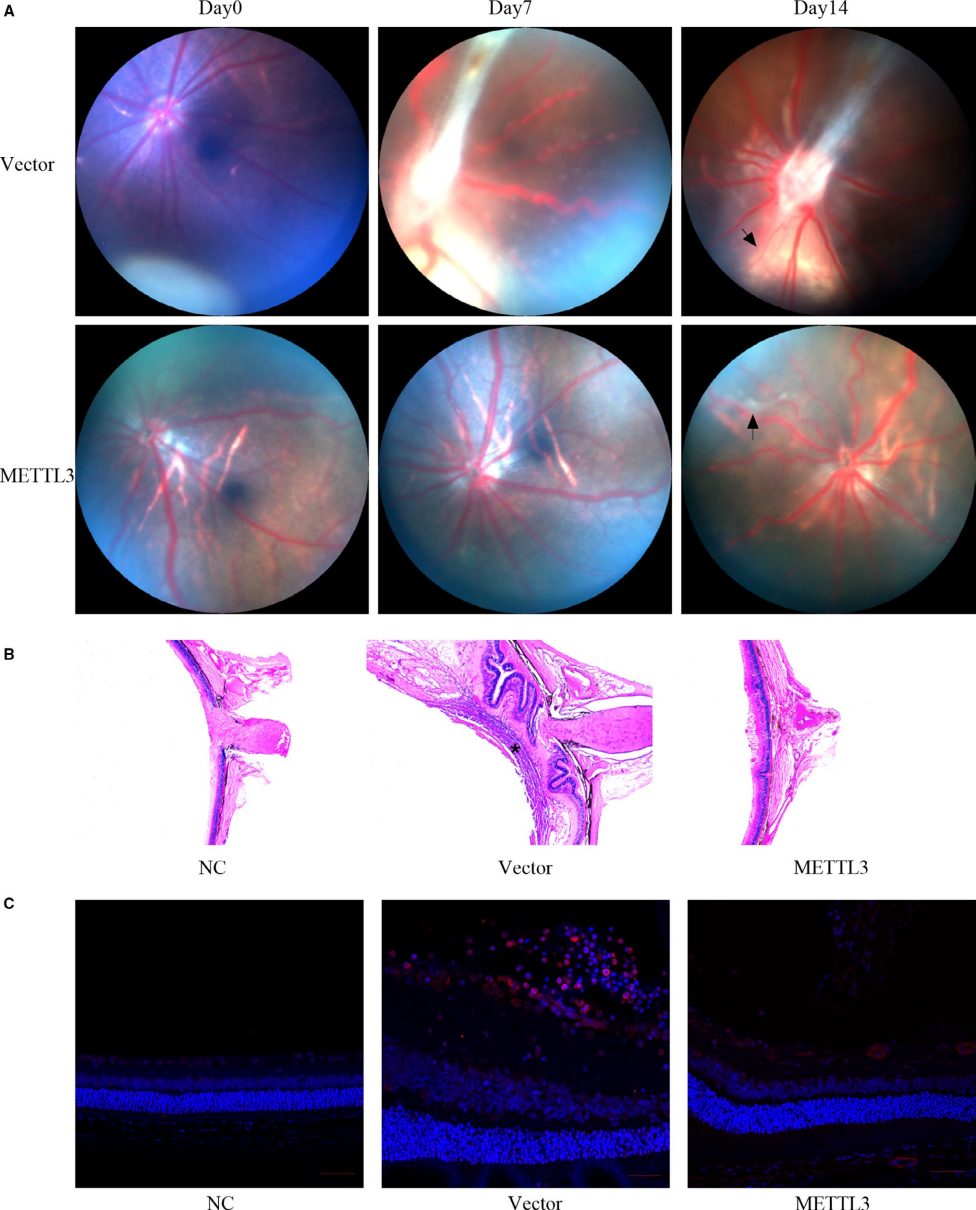
Comparison of fundus features and retinal histopathology between rats injected with vector cells and METTL3‐overexpressing cells. A, Photographs of the rat fundus at 0, 7 and 14 days. In images at day14, the black arrow in vector group indicates retinal detachment, and the black arrow in METTL3 overexpression group represents pre‐retinal proliferation. B, H&E staining of eyeball sections. The asterisk in the vector group indicates a newly formed membrane on the surface of the retina, while only little exudate was found in front of the retina in the METTL3 overexpression group. C, Immunofluorescence staining of αSMA in the vector group and METTL3 overexpression group. (n = 6)

PBS, cell suspension of the vector group or the METTL3‐overexpression group was injected into vitreous cavities of BN rats. The development of PVR in each injected eye was observed intuitively at fixed intervals. On day 7 after injection, all rats injected with vector cells had been triggered into the PVR process, with intravitreal proliferation observed in 7 rats (Stage 1) and pre‐retinal proliferation accompanied with punctate infiltration detected in 11 rats (Stage 2), and tractional detachment of the retina in 1 rat (Stage 3). By contrast, among the rats injected with METTL3‐overexpressing cells, 13 had not yet started the PVR process (Stage 0). 4 showed intravitreal proliferation (Stage 1), and 3 showed pre‐retinal proliferation (Stage 2). The difference in PVR development between the two groups was statistically significant (*P* <.01) (Table 2). However, on day 14 in vector group, 74% (14 out of 19) rats reached Stage 3 of PVR, and only 5 rats stayed at Stage 2. By contrast, on day 14 in the METTL3‐overexpression group, all rats initiated the PVR process, however, only 20% (4 out of 20) rats reached Stage 3, while 6 and 10 rats remained at Stage 1 and Stage 2 respectively. The difference in PVR development between the two groups was statistically significant (*P* <.01) (Table 2). No pathological conditions were observed in rats injected with PBS. According to Table 2, we selected rats representing the median and the fundus condition was presented by the fundus photograph (Figure 5A).

**TABLE 2:** PVR classification of rat eyes injected with METTL3‐overexpression cells and control cells

	D7			D14		
	NC	M3	*P*	NC	M3	*P*
Stage 0	0	13	<0.01	0	0	<0.01
Stage 1	7	4		0	6	
Stage2	11	3		5	10	
Stage3	1	0		14	4	

To identify the pathological features of rat retinas in three groups, all rats were sacrificed on day 14 and their eyeballs were carefully collected. The results of H&E staining showed that in the vector group, the retina was subjected to a centripetal pulling force, leading to the shrinkage and detachment of the retina accompanied with swelling and thickening. (Figure 5B). Additionally, a dense membrane was observed on the surface of the retina (Figure 5B, asterisk), with a large number of cellular components. By contrast, the METTL3 overexpression group showed only a slight shrinkage and a small amount of exudate on the retinal surface (Figure 5B). To verify the existence of myofibroblasts in both groups, we performed immunofluorescence staining of αSMA and found that αSMA positively expressed in the PVR membrane of the vector group, while less αSMA‐specific staining was detected on the surface of the retina in the METTL3 overexpression group (Figure 5C).

### 3.7 METTL3 knockdown facilitated cell proliferation and EMT of ARPE‐19 cells

**FIGURE 6:**
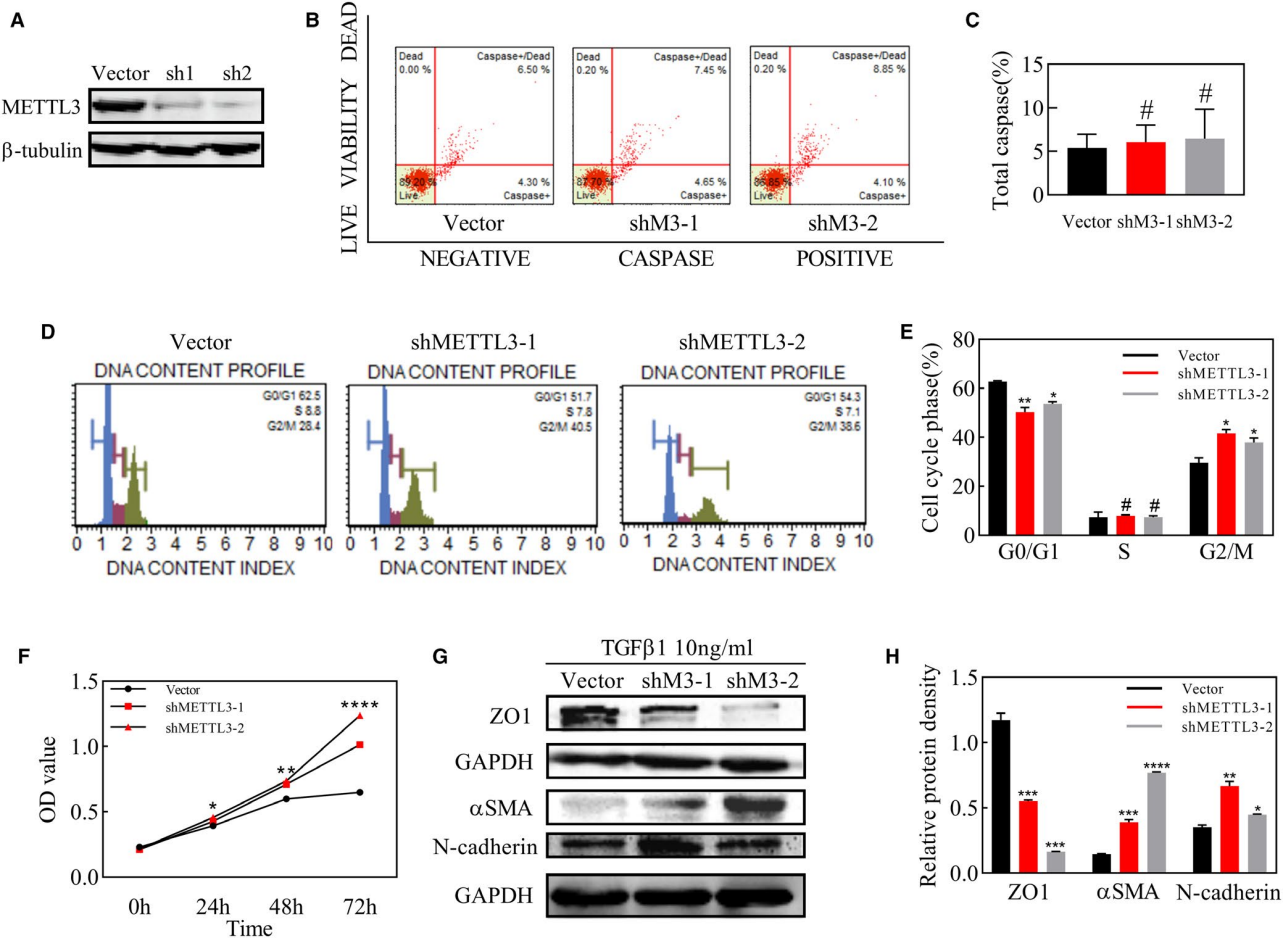
Effects of METTL3 knockdown on apoptosis, cell cycle, proliferation and TGFβ1‐induced EMT of ARPE‐19 cells. A, Western blot analysis of cells transformed with lentivirus of vector and two METTL3 shRNA and screened with puro for 48 h. B, Flow cytometry analysis of cell apoptosis at 24 h after the seeding of ARPE‐19 cells. C, Statistical analysis of the proportion of total caspase. D, Flow cytometry analysis of cell cycle at 24 h after the seeding of ARPE‐19 cells. E, Statistical analysis of the proportion of cells in each stage of the cell cycle including G0/G1, S and G2/M. F, Optical density (OD) values of ARPE‐19 cells in the MTT assay at 0, 24, 48 and 72 h after seeding. G, Western blot analysis of the epithelial marker ZO‐1 and mesenchymal markers, N‐cadherin and αSMA, after treatment with TGFβ for 48 h. H, Statistical analysis of the relative protein density, which was normalized to GAPDH. Data represent mean ± SD (n = 3). Statistical significance was determined by one‐way ANOVA, followed by Bonferroni's post hoc test for multiple comparisons (*P <.05, **P <.01, ***P <.001, ****P <.0001, #*P* >.05)

As METTL3 overexpression suppressed cell proliferation and TGFβ1‐induced EMT, we next investigated whether knockdown of METTL3 could facilitate proliferation and EMT in ARPE‐19 cells. We down‐regulated the expression of METTL3 effectively with 2 separate, specific shRNAs (Figure 6A). Apoptosis assay showed no significance between vector group and the other two groups transfected with METTL3 shRNAs, indicating that the down‐regulation of METTL3 did not induce cell toxicity (Figure 6B, 6C). METTL3 knockdown significantly accelerated the proliferation of ARPE‐19 cells in 24h, 48h and 72h after cells were seeded in the 96‐well plate (Figure 6F). Meanwhile, we found in cell cycle assay that knockdown of METTL3 reduced the proportion of cells in G0/G1 phase, while the proportion of cells in G2/M phase increased (Figure 6D,E). We further observed whether loss of METTL3 had effect on TGFβ1‐induced EMT in ARPE‐19 cells, and the results of western blot suggested that mesenchymal markers including a‐SMA and N‐cadherin were up‐regulated and epithelial marker ZO‐1 was strikingly down‐regulated in both groups transfected with METTL3 shRNAs (Figure 6G,H).

## 4 DISCUSSION

In this study, we first observed in human tissues that METTL3 expression decreased in PVR membranes compared with normal RPE layer, and we further verified in ARPE‐19 cells that total amount of m6A as well as the expression of METTL3 was down‐regulated after the induction of EMT. In the further exploration of the regulation mechanism of METTL3 on EMT in vitro, we uncovered that METTL3 overexpression inhibited cell proliferation and migration as well as the expression of EMT markers via wnt/β‐catenin pathway. Finally, it was elucidated in vivo that METTL3 delayed the initiation and development of PVR, which reinforced our findings in vitro.

RPE cells are the dominant cellular component in PVR membranes,^35^ and we found both in ERMs and SRMs that METTL3 positively expressed in RPE cells. However, RPE cells in the PVR membranes have already gone through detachment from the basement membrane and migration, and our results further elucidated that METTL3 expression was down‐regulated in PVR membranes including those migrated RPE cells compared with in‐situ RPE layer from normal individuals. αSMA is a marker of myofibroblasts, indicating the RPE cells have undergone EMT process, however, co‐staining of αSMA and METTL3 showed few overlapping areas of fluorescence, and this phenomenon was further quantified by qRT‐PCR that there was a negative correlation between the expression of αSMA and METTL3 in PVR membranes. In all, it appears that METTL3 expression decreased after the occurrence of PVR. METTL3 is a methyltransferase modulating m6A modification in mRNA, however, according to the current research, several biological processes including cellular phenotypic transformation are accompanied with alteration in the m6A modification.^23^, ^36^ Here, we showed that the abundance of m6A in ARPE‐19 cells decreased after the induction of EMT. However, the abundance of m6A synchronously depends on the dynamic regulation of methyltransferases and demethylases,^37^ among which the expression of METTL3 was strikingly down‐regulated at both the transcript and protein levels.

Recent studies suggest that METTL3 influences cell apoptosis in several cancer cells.^38^, ^39^ However, in ARPE‐19 cells, whether METTL3 overexpression or knockdown had no significant effect on cell apoptosis, although METTL3 overexpression inhibited cell proliferation. At the same time, we noticed that METTL3 overexpression induced cell cycle arrest at G0/G1 phase by inhibiting cyclinD1 expression, which might count for the suppression of cell proliferation. Our results suggested that METTL3 exerted non‐toxic effects but drove ARPE‐19 cells to an inactive state. Under this state, we applied TGFβ1 to better simulate the environment for RPE cells exposed to the cytokines during the PVR process.^40^ PVR is thought to be an abnormal wound healing response.^35^ Our results showed that METTL3 overexpression weakened the migratory ability of ARPE‐19 cells. However, the first step for cell migration is the degradation of the basement membrane and the extracellular matrix. The matrix metalloproteinase family plays a leading role in the degradation of the extracellular matrix. In this family, type IV collagenases including MMP9, are most closely related to cell migration.^41^ Based on our data, we speculate that the reduction in the migration ability partly attributes to the reduced expression of MMP9. MMP9 and cyclinD1 are both downstream targets of wnt/β‐catenin pathway, which were down‐regulated by METTL3 overexpression, so we further explored if the inhibition effect of METTL3 overexpression worked through regulating wnt/β‐catenin pathway. It came out that METTL3 suppressed the expression of several proteins belonging to wnt/β‐catenin pathway, among which β‐catenin was up‐regulated during EMT process. Furthermore, we found that SKL2001, the angonist of wnt/β‐catenin pathyway, abolished the inhibitory effect of METTL3 on EMT.^8^, ^42^ Consistent with the results in vitro, we found that injection of cells overexpressing METTL3 into the vitreous cavity of rats delayed the onset and progression of PVR. This is encouraging for future clinical applications, as it may be possible to interfere with METTL3 and m6A methylation to gain time for the early treatment of PVR.

METTL3 could play different roles in the EMT of different tumour cells, and we observed an inhibitory effect of METTL3 on the EMT of ARPE‐19 cells. One reason for this diversity may be that m6A modification has diverse decisions on RNA fate, which depends on the m6A reader family YTHDFs.^43^ Nevertheless, we uncovered that YTHDF2 expression only decreased slightly after EMT, but we cannot rule out the changes in the abundance of m6A‐bound YTHDF2. Further research is needed to explain how m6A determines the fate of mRNA.

Overall, our data demonstrated for the first time that the epigenetic modification of mRNA is involved in the pathological process of PVR, and overexpression of the m6A methyltransferase METTL3 could inhibit the EMT of ARPE‐19 cells in vitro and restrain the PVR process in vivo. The study highlights a new perspective in the clinical treatment of PVR, and provides a theoretical basis for development of new drugs.

## CONFLICTS OF INTEREST

The authors confirm that there are no conflicts of interest.

## AUTHOR CONTRIBUTIONS

**Xinqi Ma:** Data curation (lead); Methodology (lead); Writing‐original draft (lead). **Chongde Long:** Data curation (equal); Methodology (equal). **Fangyu Wang:** Data curation (equal); Methodology (supporting). **Bingsheng Lou:** Formal analysis (equal); Resources (supporting). **Miner Yuan:** Data curation (supporting); Resources (equal). **Fang Duan:** Validation (supporting); Writing‐review & editing (equal). **Yao Yang:** Funding acquisition (supporting); Writing‐review & editing (supporting). **Jiaqing Li:** Resources (equal). **Xiaobing Qian:** Methodology (supporting); Resources (supporting). **Jieting Zeng:** Data curation (supporting); Methodology (supporting). **Shuibin Lin:** Methodology (supporting); Writing‐review & editing (supporting). **Huangxuan Shen:** Conceptualization (equal); Investigation (equal); Supervision (equal); Writing‐review & editing (equal). **Xiaofeng Lin:** Conceptualization (lead); Funding acquisition (lead); Project administration (lead); Supervision (equal); Validation (lead).

## Supporting information

Figure S1

## Data Availability

The data supporting the findings of this study are available from the corresponding author upon reasonable request.
