# Supplementary figures and images for: METTL3 attenuates proliferative vitreoretinopathy and epithelial‐mesenchymal transition of retinal pigment epithelial cells via wnt/β‐catenin pathway
Source: J Cell Mol Med. 2021 Mar 23;25(9):4220–34. doi: 10.1111/jcmm.16476 (PMC8093987; doi:10.1111/jcmm.16476)

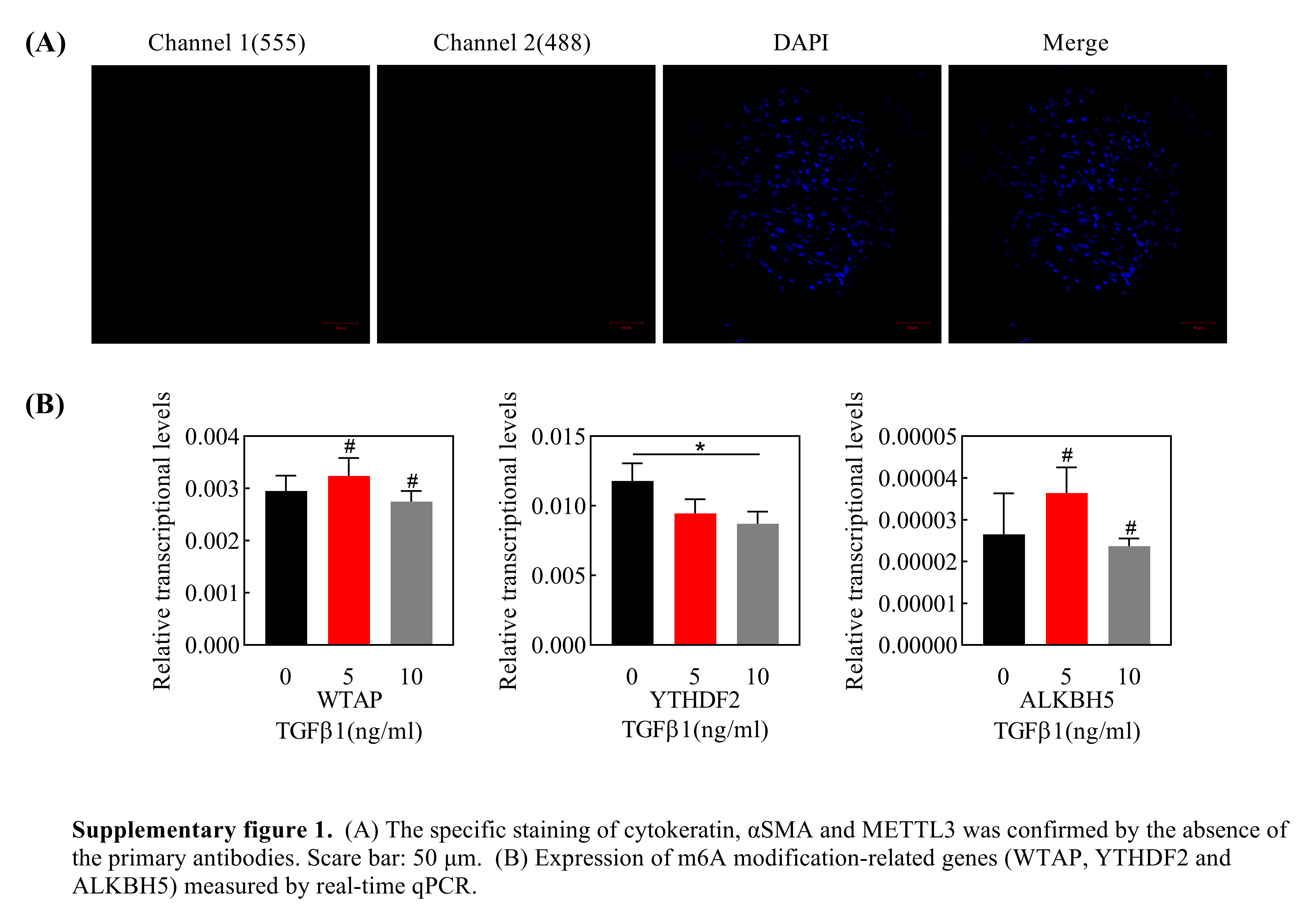

Supplement: Supplementary file 1 — Figure S1 [file JCMM-25-4220-s001.tif]
